# Supplementary material for: PeskAAS: A near-real-time, open-source monitoring and analytics system for small-scale fisheries
Source: PLoS One. 2020 Nov 13;15(11):e0234760. doi: 10.1371/journal.pone.0234760 (PMC7665685; doi:10.1371/journal.pone.0234760)
Supplement: S1 Data — (DOCX) [file pone.0234760.s001.docx]

**S1 Data.** List of species, species groups and family categories used for recording catch along with a and b parameters used to calculate weight from length measurements.

| **Species group** | **Family** | **a** | **b** |
| --- | --- | --- | --- |
| No catch | NA | NA | NA |
| Short bodied mackerel | Scombridae | 0.013 | 3.21 |
| Mackerel scad | Carangidae | 0.00783 | 3.14 |
| Jacks/Trevally/Other Scad | Carangidae | 0.0222 | 2.939 |
| Tuna/Bonito/Other Mackerel | Scombridae | 0.0164 | 3.05 |
| Fusilier | Caesionidae | 0.0124 | 3.112 |
| Sardines/pilchards | Clupeidae | 0.00262 | 3.225 |
| Garfish | Hemiramphidae | 0.00545 | 3.134 |
| Soldierfish | Holocentridae | 0.0215 | 3 |
| Long tom | Belonidae | 0.00078 | 3.205 |
| Snapper/seaperch | Lutjanidae | 0.0216 | 2.95 |
| Spinefoot | Siganidae | 0.01825 | 3 |
| Grouper | Serranidae | 0.0155 | 3.002 |
| Emperor | Lethrinidae | 0.0197 | 2.979 |
| Surgeonfish | Acanthuridae | 0.0216 | 2.988 |
| Parrotfish | Scaridae | 0.0188 | 3.026 |
| Triggerfish | Balistidae | 0.031 | 2.947 |
| Flying fish | Exocoetidae | 0.076 | 2.381 |
| Unicornfish | Acanthuridae | 0.0216 | 2.988 |
| Mojarra/Silverbelly | Gerreidae | 0.0176 | 3.059 |
| Jobfish | Lutjanidae | 0.0216 | 2.95 |
| Wrasse | Labridae | 0.01687 | 3 |
| Barracuda | Sphyraenidae | 0.0108 | 2.884 |
| Chub | Kyphosidae | 0.0179 | 3 |
| Octopus | Octopodidae | 0.018 | 3 |
| Moonfish | Menidae | 0.0229 | 3 |
| Moontail bullseye | Priacanthidae | 0.021 | 2.83 |
| Herring | Elopidae | 0.02997 | 2.80085 |
| Ponyfish | Leiogthidae | 0.02704 | 2.97961 |
| Bream | Nemipteridae | 0.015 | 2.98 |
| Blackspot sweeper | Pempheridae | 0.01331 | 3 |
| Goatfish | Mullidae | 0.0123 | 3.081 |
| Mullet | Mugilidae | 0.013 | 2.98 |
| Terapon | Terapontidae | 0.0338 | 2.939 |
| Shark | Carcharhinidae | 0.0109 | 3.012 |
| Sweetlips | Haemulidae | 0.014 | 3 |
| Moray | Muraenidae | 0.00173 | 3 |
| Sergeant | Pomacentridae | 0.00989 | 3.267 |
| Shrimp | NA | NA | NA |
| Butterflyfish | Chaetodontidae | 0.02776 | 2.97346 |
| Cardinalfish | Apogonidae | 0.0137 | 3.037 |
| Cuttlefish | Sepiidae | NA | NA |
| Javelin/Grunt | Haemulidae | 0.0187 | 3.156 |
| Sailfish | Istiophoridae | 0.0161 | 2.72 |
| Cobia | Rachycentridae | 0.00646 | 3.15 |
| Crab | NA | NA | NA |
| Dolphinfish | Coryphaenidae | 0.0229 | 2.78 |
| Bannerfish | Chaetodontidae | 0.02502 | 3 |
| Milkfish | Chanidae | 0.0204 | 3.024 |
| Threadfin | Polynemidae | 0.0102 | 3.02 |
| Cockles | Cardiidae | NA | NA |
| Remora | Echeneidae | 0.00102 | 3.29 |
| Tripodfish | Triacanthidae | 0.01866 | 3 |
| Wolf herring | Chirocentridae | 0.00509 | 2.99 |
| Stingrays | Myliobatoidae | 0.00869 | 3 |
| Sicklefish | Drepaneidae | 0.0167 | 3.19 |
| Other | NA | 0.0102 | 3.02 |
| Unknown | NA | 0.0102 | 3.02 |
