# Supplementary material for: PeskAAS: A near-real-time, open-source monitoring and analytics system for small-scale fisheries
Source: PLoS One. 2020 Nov 13;15(11):e0234760. doi: 10.1371/journal.pone.0234760 (PMC7665685; doi:10.1371/journal.pone.0234760)

**S2 Data.** MySQL relational database schema with cascading foreign keys shown by dashed arrows. Figure generated in MySQL Workbench (Oracle Corp.).


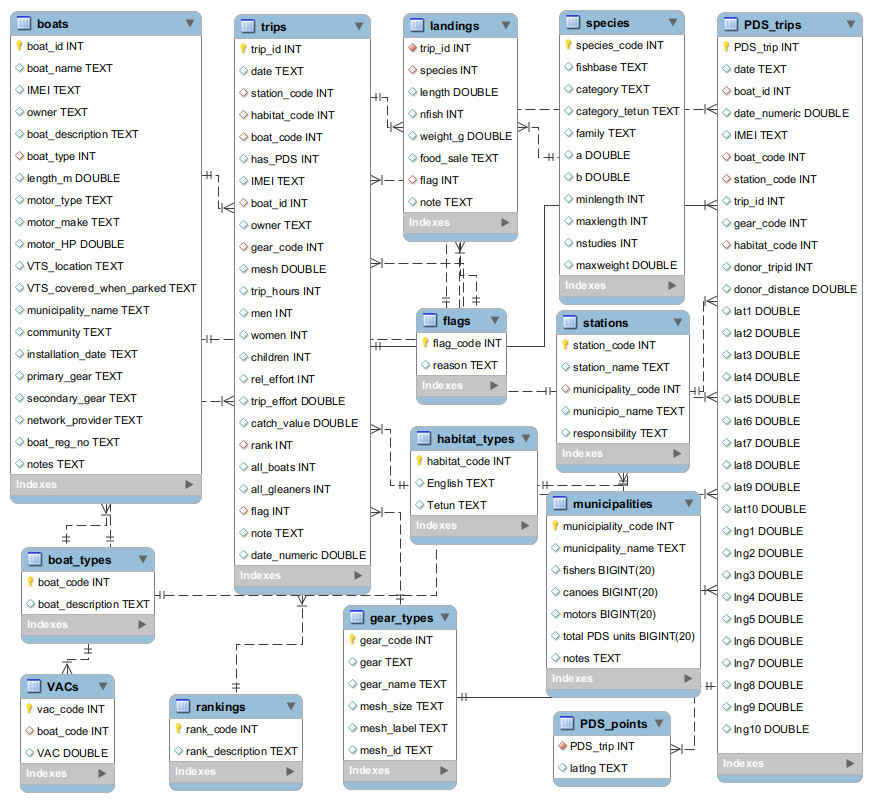

Supplement: S2 Data — Figure generated in MySQL Workbench (Oracle Corp.). (DOCX) [file pone.0234760.s002.docx]
